# A case-control study of *GST *polymorphisms and arsenic related skin lesions

**DOI:** 10.1186/1476-069X-6-5

**Published:** 2007-02-06

**Authors:** Kathleen M McCarty, Louise Ryan, E Andres Houseman, Paige L Williams, David P Miller, Quazi Quamruzzaman, Mahmuder Rahman, Golam Mahiuddin, Thomas Smith, Ernesto Gonzalez, Li Su, David C Christiani

**Affiliations:** 1 Yale University School of Medicine, Epidemiology and Public Health, Division of Environmental Health Sciences, New Haven, CT, USA; 2 Harvard School of Public Health Department of Environmental Health, Boston, MA, USA; 3 Harvard School of Public Health Department of Biostatistics, Boston, MA, USA; 4 Dhaka Community Hospital, Dhaka, Bangladesh; 5 Massachusetts General Hospital, Boston MA, USA

## Abstract

**Background:**

Polymorphisms in *GSTT1*, *GSTM1 *and *GSTP1 *impact detoxification of carcinogens by GSTs and have been reported to increase susceptibility to environmentally related health outcomes. Individual factors in arsenic biotransformation may influence disease susceptibility. GST activity is involved in the metabolism of endogenous and exogenous compounds, including catalyzing the formation of arsenic-GSH conjugates.

**Methods:**

We investigated whether polymorphisms in *GSTT1*, *GSTP1 *and *GSTM1 *were associated with risk of skin lesions and whether these polymorphisms modify the relationship between drinking water arsenic exposure and skin lesions in a case control study of 1200 subjects frequency matched on age and gender in community clinics in Pabna, Bangladesh in 2001–2002.

**Results and discussion:**

*GSTT1 *homozygous *wildtype *status was associated with increased odds of skin lesions compared to the null status (OR1.56 95% CI 1.10–2.19). The *GSTP1 GG *polymorphism was associated with greater odds of skin lesions compared to *GSTP1 AA*, (OR 1.86 (95%CI 1.15–3.00). No evidence of effect modification by *GSTT1*, *GSTM1 *or *GSTP1 *polymorphisms on the association between arsenic exposure and skin lesions was detected.

**Conclusion:**

*GSTT1 wildtype *and *GSTP1 GG *are associated with increased risk of skin lesions.

## Background

Arsenic exposure through drinking water is a global problem, and has reached crisis status in Bangladesh [1-5]. A well established exposure-response relationship exists between arsenic level of drinking water and skin lesions[6,7]. Skin lesions are considered one of the most distinctive endpoints of chronic arsenic exposure[8].

It has been proposed that there are differences in susceptibility to arsenic due to individual genetic variability in biotransformation of the metal[9]. Polymorphisms in *GST *genes have been associated with susceptibility to a range of diseases, and *GST *polymorphisms alone and in concert with environmental exposures are associated with disease outcomes and behavior of several enzymes [10-12]. Glutathione S-transferases (GST) are a superfamily of enzymes that are key in the detoxification step of Phase II metabolism, usually by catalyzing the conjugation of reduced glutathione (GSH) into hydrophobic and electrophilic compounds along with other Phase II enzymes [10-12]. *In vivo *studies have shown that GSH serves as a reducing agent required for the reduction of arsenate to arsenite[13]. GSH also serves as a reducing agent in the methylation of arsenic from arsenite to MMM (V) and from MMA (III) to DMA (V) [13]. GST activity is involved in the metabolism of endogenous and exogenous compounds, including catalyzing the formation of arsenic-GSH conjugates[13,14]. Animal data had demonstrated that these conjugates are transported by multidrug resistant protein transporters (MRP) from the liver to the bile [14-17]. Glutathione and related enzymes are also involved in cellular protection against reactive oxygen species (ROS) [11,12]. Chronic arsenic exposure has been shown to alter glutathione metabolism and cellular redox status and maintenance of cellular redox state may have an important role in arsenic related pathology[14,18,19].

The biologic control of GST enzymes is multifaceted in that they demonstrate specific patterns of expression that depend on sex, age, tissue, and species and vary between individuals [11,20]. *GSTM1*, *GSTT1 *and *GSTP1 *are members of the Mu (μ), Theta (θ), and Pi (π) classes respectively[10]. Polymorphisms in *GSTT1*, *GSTM1 *and *GSTP1 *alone or in concert with environmental exposures may be associated with increased susceptibility to environmentally related diseases such as cancer and other clinical outcomes[10,12]. The *GSTT1 null *and *GSTM1 null *genotypes are deletion polymorphisms and have no θ or μ-glutathione S transferase activity respectively. The *GSTP1 *polymorphism is a single base pair substitution where adenine is replaced by guanine resulting in an amino acid change in which isoleucine (I_105_) is replaced by valine (V_105_), possibly resulting in lower enzyme activity[21,22]. At higher arsenic exposure, increased GST activity may be associated with saturation of MRP transporters allowing increased tissue accumulation[14]. We hypothesized that elevated glutathione-S-transferase activity, specifically activity of glutathione-μ,θ, and π transferases, may be associated with increased risk of skin lesions.

We investigated the relationship between *GSTT1, GSTM1*, and *GSTP1 *polymorphisms and skin lesions. In addition, we assessed possible effect-modification by *GST *genotypes in modifying the risk of arsenic related skin lesions using well water arsenic concentration to estimate exposure.

## Methods

### Study population

This study was conducted in the Pabna district of Bangladesh, located north of Dhaka on the Pabna (Ganges) River. Pabna was chosen for the following reasons: elevated arsenic was suspected in some of the region's villages due to proximity to the River; Dhaka Community Hospital (DCH) has a well established clinic network in the area; and Pabna is representative of socioeconomic status of much of non-urban Bangladesh. Eligible cases were Pabna residents, at least 16 years of age, with one or more type of skin lesion: diffuse/spotted melanosis, diffuse/spotted keratosis, hyperkeratosis, or leukomelanosis. One physician made the diagnosis, and treatment was provided at DCH when necessary. Controls were healthy individuals diagnosed as free of skin lesions and arsenic related disease randomly selected in a 1:1 ratio from Pabna, age of at least 16 years, living in the same village as cases but not sharing a tube well. Controls were also frequency matched to cases based on gender and age (+/- 3 years). To ensure heterogeneity of exposure and to prevent overmatching on exposure, controls were further selected so as to ensure that 80% were in "low-exposure" arsenic (<50 μg/l) communities and 20% were from suspected "high exposure" (≥50 μg/l) areas. This last ensured that the exposure distribution among controls matched that which has been reported for the Pabna region as a whole[23].

Initial measurements of well arsenic levels were made with Merck field test kits[24]. Individuals found to have arsenic exposure greater than 50 μg/l were advised of alternative drinking water sources. The participation rate was 98.0%; a total of 24 subjects from 1224 declined to participate. Cases and controls had similar reasons for refusal. The population is ethnically homogenous, and similar to the population of Bangla (West Bengal), India. Informed consent was obtained from all study participants. The study protocol was approved by the Institutional Review Boards at Dhaka Community Hospital, Bangladesh and Harvard School of Public Health Boston, MA, USA.

### Interviews and sample collection

In 2001–2002, 1200 subjects were recruited. Physicians, blinded to exposure status, examined potential cases and controls. Trained interviewers administered the questionnaire regarding exposure, lifestyle factors, and collected individual well water samples. Data were collected on liters of water/liquid ingested per day, disease history, residential history including identification of the primary water source (tube well), years of use, and use of a previous tube well.

Collection of well water samples was designed to minimize bias. Due to the fact that "high" exposure (≥50 μgAs/l) wells were often painted red and "low" exposure wells (<50 μgAs/l) were often painted green, the field team would have known if they had some indication as to whether the well was above or below 50 μg As/l. However, the field team did not know the arsenic concentration of the well at the time the subject was examined and interviewed, a procedure similar to a study in West Bengal [25].

Additionally, it has been documented that wells are often mislabeled[26]. Thus, the field team was blind to the true exposure level of the subjects at the time case status was determined. Water samples were analyzed in the United States and the field team received results after subjects were enrolled.

Upon collection of each 100 ml water sample two drops (0.2 ml) of pure nitric acid was added. The samples were stored in a cooler before storage in a 4°C refrigerated room. Analysis of each sample for arsenic concentration was completed using Environmental Protection Agency (EPA) method 200.8 with Inductively Coupled Plasma Mass Spectroscopy (ICP-MS) (Environmental Laboratory Services, North Syracuse, New York)[27]. The method limit of detection was 1 μg As/l.

Two 10 ml EDTA tubes were used to collect blood and were stored in a cooler on ice until processed with cell lysis solution. Samples were sent to the Molecular Epidemiology Laboratory at Harvard School of Public Health for DNA extraction and genotyping.

### Genotyping

DNA samples were stored at -80°C. The *GSTM1 *and *GSTT1 *genetic polymorphisms were evaluated using a previously described multiplex PCR technique[28]. *GSTP1 *polymorphism was genotyped by the 5' nuclease assay (TaqMan) using the ABI Prism 7900HT Sequence Detection System (Applied Biosystems, Foster City, CA). The primers, probes, and reaction conditions are available upon request. Genotyping for *GSTT1 *and *GSTM1 *was completed for 1062 subjects, and genotyping for *GSTP1 *was completed for 1101 subjects. Laboratory personnel were blinded to case status, and a random 5% of the samples were repeated to validate genotyping procedures. Two authors independently reviewed all results with 100% concordance.

### Statistical analysis

Analysis was restricted to subjects who reported using the same well greater than 6 months to minimize potential for temporal variability in well arsenic concentration. Since subjects were frequency matched by age and sex, these variables were included in all regression models[29,30]. Arsenic concentration and volume of liquid consumed per day were not combined as a dose variable, since liquid volume included juice, milk, soup, tea, and water. Data exploration using generalized additive models (GAMs), implemented in R (version 1.8.1), suggested that the log-odds of case status varied linearly with the arsenic concentration of well water; consequently untransformed arsenic concentration was used as a continuous predictor of case status. The models suggested that the log-odds of case status had a nonmonotonic relationship with BMI; therefore a quadratic term for BMI was included. To facilitate numerical stability, both linear and quadratic terms for BMI were centered at the median BMI value, 19.1. Consolidated categories for educational status and age were established.

Univariate analyses were performed to describe population characteristics and to identify possible data errors and/or outliers. Continuous variables were summarized using means, medians, standard deviations and ranges, while categorical variables were described using percentages. Bivariate analyses (chi-squared tests or t-tests, as appropriate) were conducted explore differences between cases and controls prior to multivariate modeling. The frequency distribution of the *GSTT1*, and *GSTM1 *polymorphisms were tested among controls to ensure Hardy-Weinberg equilibrium. The heterozygote and homozygote variant were not combined for *GSTP1*.

Multiple unconditional logistic regression was used to evaluate the associations between arsenic exposure in drinking water on case status. Odds Ratios were obtained from the regression models, as were their 95% confidence intervals. Regression models were fit using Statistical Analysis Systems (SAS Institute Inc, Cary NC) version 8.2.

Two different analyses were done to investigate: 1) the associations of *GST *polymorphisms with skin lesions, adjusting for well arsenic concentration and 2) the modification of the relationship between well arsenic concentration and skin lesions by GST polymorphisms. The joint effects (interaction) of arsenic exposure and each of the genes were evaluated in additive and multiplicative models. Categorical variables were created for measures of arsenic exposure: drinking water arsenic concentration <50 μg/l and ≥50 μg/l. The deletion polymorphisms *GSTT1 *and *GSTM1*, were modeled as homozygous wildtype and heterozygotes compared to homozygote null.*GSTP1 *was modeled to determine whether the heterozygote variant and the homozygote variant might be associated with risk of developing skin lesions compared to wildtype. Variables were created for each combination of arsenic exposure and genotype, with the reference category being low arsenic exposure and either *GSTT1 null*, *GSTM1 null *or *GSTP1 wildtype AA*. Adjusted ORs and 95% CIs were evaluated for deviation from the expected null value on the additive or multiplicative scale. Interaction Contrast Ratios (ICR) and Bootstrap Percentile Method 1 (BP1) 95% CIs were calculated to quantify departure from additivity[31]. Synergy on the additive scale is implied by ICRs greater than zero. ICRs of zero imply no additive effects on the additive scale. Antagonism on the additive scale is implied by ICRs less than zero[32]. To estimate interaction on the multiplicative scale adjusted ORs and 95% CIs were estimated in separate logistic regression models with interaction terms for each genotype and arsenic exposure as a continuous variable. Likelihood ratio tests were conducted comparing adjusted models with main effects for *GSTT1, GSTM1 *or *GSTP1 *and arsenic exposure and an interaction term compared to the same model excluding the interaction term.

**Figure 1:**
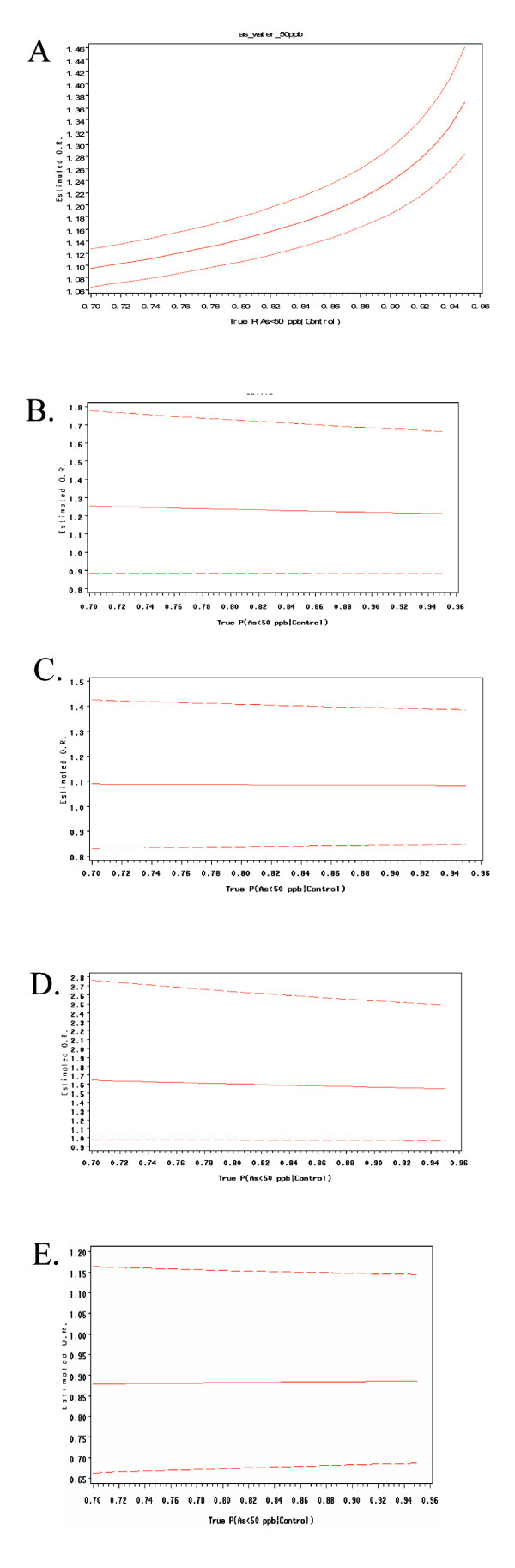
**(A) **Results from a sensitivity analysis conducted to determine the stability of the main effect of tube well arsenic concentration in 50 ug/L intervals on the odds ratio for developing skin lesions. (B) Results from a sensitivity analysis conducted to determine the stability of the main effect of *GSTT1 *genotype on the odds ratio for developing skin lesions. (C). Results from a sensitivity analysis conducted to determine the stability of the main effect of *GSTM1 *on the odds for developing skin lesions. (D) Results from a sensitivity analysis conducted to determine the stability of the main effect of *GSTP1 GG *allele on the odds for developing skin lesions. (E) Results from a sensitivity analysis conducted to determine the stability of the main effect of *GSTP1 AG *allele on the odds for developing skin lesions. The sensitivity analysis evaluated the influence of control selection allowing for 70–95% of the controls being selected from tube wells containing less than 50 ug/L. The black line represents the sampling design employed in this study which assumed that the 80% of the tube wells in Pabna contained arsenic concentrations below 50 ug/L. As the percentage of controls with arsenic concentrations below 50 ug/L increases, the OR for skin lesions increases associated with each 50 μg/l increase in tube well arsenic. The X-axis is the percentage of controls selected from areas suspected to have well water arsenic concentration less than 50 μg/l. Odds ratios and 95% CI are graphed to show the stability of the effect estimates as the percentage of controls from low exposure areas are varied in the logistic regression model.

Sensitivity analysis was conducted by varying weights of controls selected having a well concentration less than 50 μg/l in a weighted logistic regression analysis as described previously[33]. Briefly, this method was used to determine whether the percentage of controls selected from suspected high and low arsenic areas impacted the stability of the ORs of all of the covariates in the regression models. The weighting varied between 70%–95% of controls with suspected low exposure (<50 μg As/l) and 30% -5% of controls with suspected high exposure (≥50 μg As/l). Results can be examined graphically in Figure 1.

## Results

Demographic data are presented in Table 1. Of 592 cases, diffuse melanosis accounted for 31.7% of the cases (n = 377), followed by leukomelanosis (n = 342), spotted melanosis (n = 145), diffuse keratosis (n = 117), spotted keratosis (n = 73) and hyperkeratosis (n = 40). Subjects often had multiple types of lesions. Cases and controls were not significantly different in terms of age, BMI or gender. Controls reported using their current well for a longer duration (p = 0.02), and, conversely, cases reported a higher frequency of previous well use (p = 0.007). As expected, cases had significantly higher well arsenic concentrations (p < 0.0001). No significant difference in total liquid consumption was observed. A higher proportion of cases reported betel nut use (p = 0.007), however there was no significant difference between cases and controls in terms of years of betel nut use (p = 0.78) and number of betel nuts chewed daily (p = 0.53). A higher proportion of cases reported chewing tobacco use (p < 0.0001), however there was no difference in years of chewing tobacco use. Conversely, a higher proportion of controls reported current cigarette use (p = 0.03) and ever having smoked (p = 0.0007). Educational status was not significantly different between cases and controls. Table 1 shows the genotypes frequencies for *GSTM1 *and *GSTT1 *and allele frequencies for *GSTP1 *among cases and control subjects. The crude frequencies of the *GST *SNPs were not significantly different between cases and controls.

**Table 1:** Characteristics of Skin-Lesion Cases and Population-Based Controls in Pabna, Bangladesh

		**Controls**		**Cases**		
Diffuse Melanosis				n = 377		
Leukomelanosis				n = 342		
Spotted Melanosis				n = 145		
Diffuse Keratosis				n = 117		
Spotted Keratosis				n = 73		
Hyperkeratosis				n = 40		
**Mean Age in yrs (SD)**		33.7 (12.6)	n = 597	33.9 (12.7)	n = 592	P = 0.58
**Mean Body Mass Index (kg/m2) (SD)**		20.4 (3.1)	n = 597	20.1 (3.1)	n- = 592	P = 0.70
**% Male**		60.3%	n = 360	60.3%	n = 357	P = 0.91
**Mean Duration of Present well use(yrs) (SD)**		10.1 (9.0)	n = 592	8.0 (7.2)	n = 592	P = 0.02
**% Reported a Previous Well**		2.84%	n = 17	7.77%	n = 45	P < 0.0001
**Mean As concentration of current well (μg/l) (SD)**		66.2 (149.6)	n = 595	232.8 (315.7)	n = 592	P < 0.0001
**Mean Daily Total Water/Liquid consumption (L) (SD)**		3.8 (1.2)	n = 595	3.7 (1.1)	n = 592	P = 0.84
**% Ever used Betel nuts**		24.3%	n = 145	27.7%	n = 164	P = 0.007
**Mean Years of Betel nut Use (SD)**		10.8 (8.9)	n = 143	11.0 (9.5)	n = 160	P = 0.78
**Mean Number of Betel nuts chewed per day (SD)**		5.6 (3.6)	n = 158	5.7 (3.8)	n = 149	P = 0.53
**% Chew tobacco leaves**		16.4%	n = 587	17.1%	n = 590	P < 0.0001
**Mean Years of Tobacco leaves chewed (SD)**		9.9 (9.1)	n = 95	10.9 (9.4)	n = 95	P = 1.0
**% Smokes Cigarettes Currently**		30.5%	n = 597	26.7%	n = 592	P = 0.03
**% Ever Smoked**		31.0%	n = 597	28.7%	n = 592	P = 0.0007
**Education Level**		n = 597		N = 592		P = 0.80
**% Illiterate**		17.4%	n = 104	22.9%	n = 136	
**% Literate (incomplete Primary Education)**		23.8%	n = 142	29.4%	n = 174	
**%Completed Primary Education**		11.7%	n = 79	11.8%	n = 70	
**% Completed Middle School Education**		31.9%	n = 191	23.5%	n = 139	
**%Completed Secondary Education or More**		13.6%	n = 81	26.0%	n = 154	
**Polymorphims:**						
***GSTT1***						P = 0.07
**Null**	**17.9%**	18.9%	n = 112	16.9%	n = 100	
**Wildtpe**	**82.1%**	81.1%	n = 482	83.1%	n = 492	
***GSTM1***						P = 0.72
**Null**	**41.1%**	41.1%	n = 244	41.0%	n = 243	
**Wildtype**	**58.9%**	58.9%	n = 350	59.0%	n = 349	
***GSTP1***						P = 0.07
**AA**	**53.8%**	53.5%	n = 313	54.1%	n = 318	
**AG**	**38.7%**	40.3%	n = 236	37.1%	n = 218	
**GG**	**7.5%**	6.2%	N = 36	8.8%	n = 52	

The main effects for each genotype on skin lesions adjusted for well arsenic concentration was shown in Table 2. The *GSTT1 wildtype *gene carried a higher risk of skin lesions compared to the *null *polymorphism (OR = 1.56 (95%CI 1.10–2.19). Compared to the reference category *GSTP1 *AA, the *GG *genotype had a 86% increase odds of skin lesions (OR = 1.86 95%CI 1.15–3.00). We were not able to detect a significant association with *GSTM1 *genotype and skin lesions. All models were adjusted for well As level, L/day of total liquid, previous well use, age, sex, education, BMI, chewing tobacco, betel nut use, and smoking status.

**Table 2:** Crude and Adjusted Odds Ratios and 95% CIs for Skin Lesions Predicted by *GST *Polymorphisms.

	**Drinking Water Arsenic Concentrations**			
	
	**Crude Model 1**		**Adjusted Model 1**	
**Genotype**	**OR (95% CI)**	**p-value**	**OR (95% CI)**	**p-value**
***GSTP1***				
***AA***	1.0		1.0	
***AG***	0.90 (0.69–1.17)	0.44	0.89 (0.68–1.17)	0.41
***GG***	1.79 (1.12–2.88)	0.02	1.86 (1.15–3.00)	0.01
***GSTT1 null***	1.0		1.0	
***GSTT1 wildtype***	1.52 (1.08–2.13)	0.02	1.56 (1.10–2.19)	0.01
***GSTM1 null***	1.0		1.0	
***GSTM1 wildtype***	1.0 (0.78–1.29)	0.98	0.99 (0.77–1.28)	0.95

Crude Models adjusted for As concentration of well or As concentration of well water. Model 1 adjusted for L/day of water, well As concentration, age, gender, education, BMI, chewing tobacco, betel nut use, and smoking status

Adjusted ORs for the joint effects of well arsenic exposure and each *GST *polymorphisms on skin lesions are shown in Table 3. Reference categories were low arsenic exposure and *GSTM1 null*, *GSTT1 null *or *GSTP1 AA*, respectively. When the results were stratified on exposure, individuals with the *GSTT1 wildtype *had a higher risk of lesions in the low (OR = 1.62, 95% CI 1.06–2.49) exposure group as well as the high exposure group, though the relationship was not significant at the high arsenic exposure level (OR = 1.15 95%CI 0.68–1.94). There is little evidence of effect modification by the *GSTM1 *genotype. In the low exposure strata, individuals with the *GSTP1 GG *genotype had a higher risk of skin lesions (OR = 2.32, 1.31–4.09) than those individuals with the *AA *genotype. In the high exposure strata there was no significant effect of the *GSTP1 *genotype. When the analysis is stratified by genotype for *GSTP1*, results were difficult to interpret due to the insufficient numbers in high exposure and *GSTP1 GG *category. Likelihood Ratio Tests (LRT) were not statistically significant (*GSTT1 *0.88, *GSTM1 *0.96, and *GSTP1 *0.21).

**Table 3:** Joint effects of *GST *genotype and drinking water level of arsenic on Case Status

				Overall Joint Effects				Joint Effects Stratified on Exposure		Joint Effects Stratified on Genotype	
	As Level of Well	Controls (N)	Cases (N)	OR (95%CI)	p-value	ICR	95%CL	OR (95%CI)	p-value	OR (95%CI)	p-value

*GSTT1*											
Null	low	85	52	1.0				1.0		1.0	
Wildtpe	low	376	272	1.62 (1.06–2.49)	0.03			1.62 (1.06–2.49)	0.03	1.0	
null	high	27	48	3.16 (1.71–5.85)	0.0002			1.0		3.16 (1.71–5.85)	0.0002
Wildtpe	high	106	222	3.64 (2.31–5.74)	<0.0001			1.15 (0.68–1.94)	0.60	2.24 (1.69–2.98)	<0.0001
						0.94	(-2.08–1.83)				
*GSTM1*											
Null	low	189	126	1.0				1.0		1.0	
Wildtype	low	272	198	1.02 (0.74–1.40)	0.91			1.02 (0.74–1.40)	0.91	1.0	
Null	high	55	117	2.62 (1.77–3.90)	<0.0001			1.0		2.62 (1.77–3.90)	<0.0001
Wildtype	high	78	151	2.24 (1.56–3.22)	<0.0001			0.85 (0.57–1.29)	0.85	2.20 (1.57–3.09)	<0.0001
						1.02	(-2.40–1.57)				
*GSTP1*											
AA	low	246	180	1.0				1.0		1.0	
AG	low	184	110	0.81 (0.58–1.14)	0.23			0.81 (0.58–1.14)	0.23	1.0	
GG	low	26	32	2.32 (1.31–4.09)	0.004			2.32 (1.31–4.09)	0.004	1.0	
AA	high	67	138	2.27 (1.60–3.22)	<0.0001			1.0		2.27 (1.60–3.22)	<0.0001
AG	high	52	108	2.49 (1.69–3.68)	<0.0001	0.85	(-2.13–1.51)	1.10 (0.72–1.69)	0.67	3.06 (2.01–4.66)	<0.0001
GG	high	10	20	2.38 (1.11–5.13)	0.03	1.68	(-2.15–1.83)	1.04 (0.48–2.31)	0.90	1.03 (0.41–2.55)	0.95

All models adjusted for liters of liquid/day, age, gender, educational status, BMI, chewing tobacco, betel nut use, smoking status, and previous well use

Interaction Contrast Ratios (ICR)s for the joint effects of arsenic exposure and the *GST *polymorphisms are shown in Tables 3. ICRs were not significant. The results are imprecise and should be interpreted cautiously.

Results of the sensitivity analysis are described in Figure 1. The sensitivity analysis of estimates for skin lesion risk predicted by well arsenic concentration varied with the weighting of controls selected from suspected high and low arsenic areas described previously[33]. As expected, varying the percentage of controls with drinking water As exposure <50 μg/l did not bias the effect estimates for *GSTM1, GSTT1*, or *GSTP1 *for risk of skin lesions (Figure 1).

## Discussion

We found that *GSTT1 wildtype *compared to *GSTT1 null*, and *GSTP1 GG *compared to *GSTP1 AA *were associated with an increased odds of arsenic related skin lesions. While the data were suggestive of *GST *polymorphisms modifying the effect of arsenic exposure levels in drinking water on risk of skin lesions, these effects were not statistically significant.

We found that there was an increased risk of skin lesions among *GSTT1 wildtype *individuals who produce enzymes that may be associated with increased reduction of glutathione. Previous research has found GST levels increased in arsenic exposed mice and this was thought to be associated with enhanced arsenic efflux by MRP transporters[14]. While there has been no experimental evidence for *GSTT1*, increased *GST *activity is associated with enhanced arsenic efflux by MRP transporters, and it is hypothesized that transporters may become saturated at higher exposures. Saturation may result in elevated tissue accumulation of arsenic and increased risk of disease[14]. Furthermore *GSTP1 *activity may work synergistically with MRP transport of inorganic arsenic as a tri-GSH conjugate[34].

We report for the first time that the *GSTP1 GG *genotype increases the risk of arsenic related skin lesions in a population based study. In vitro studies have shown that cells that express higher levels of *GSTP1 *activity were less sensitive to arsenic trioxide induced apoptosis, than cells devoid of *GSTP1 *activity and expression[35]. *GSTP1 *has been shown to increase growth inhibition of arsenic treated cancer cells and to prevent apoptosis by inhibiting JNK and p38 kinase activity[36]. Our findings are consistent with this, in that those individuals with the polymorphism associated with lower enzyme activity may be more sensitive to the effects of arsenic. However another in vitro study noted that cancer cells were most sensitive to arsenic exposure when GSH was depleted, but that the cellular level of *GST-π *did not affect cellular sensitivity to arsenic[37]. While *GSTO1-1 *has been shown to reduce methylated arsenic intermediates, *in vitro *studies have suggested that *GSTP1 *expression may promote arsenic methylation in cancer cells [38-41]. Further study is needed to clarify the role of the *GSTP1 *Iso105Val polymorphism in arsenic metabolism and in the risk of arsenic related skin lesions.

It has been suggested that GST substrates and glutathione conjugates have the ability to induce a variety of Phase II enzymes, so that polymorphisms in *GST *may influence other chemical defense mechanisms[12]. The role of the *GSTP1 *Isoleucine (105) to Valine (105) polymorphism remains to be explained [42], as the polymorphism leading to lower enzyme activity appears to be associated with higher risk of skin lesions. In contrast, the expression of glutathione-theta-transferase through *GSTT1 wildtype *expression appears to be associated with a higher risk of disease. The impact of *GSTT1*, and *GSTP1 *polymorphisms, arsenic exposure and skin lesions and molecular mechanisms require further study.

Limitations of our study include the possibility of recall bias with regard to reported Liters of liquid consumed per day. Moreover, this measure included water as well as other beverages, resulting in possible exposure misclassification for that variable. As with all sample analysis there is a potential for measurement error. However, rigorous quality control procedures were in place for the analysis of water, as well as for DNA extraction and genotyping. In a previous study in West Bengal India, it was reported by Ghosh et al. that individuals with *GSTM1 *wildtype had significantly higher risk of arsenic-induced skin lesions (Odds Ratio, 1.73; 95% confidence interval, 1.24–2.22) [43]. This study did not observe an association between *GSTT1 *or *GSTP1 *and skin lesions in an arsenic exposed population. Frequencies of GST polymorphisms were similar between the West Bengal study and our results. Due to a much smaller sample size Ghosh et al may have had limited power to detect associations with *GSTT1 *and *GSTP1*. Additionally the West Bengal study had only 22 controls and 33 cases who were homozygote null (-/-), and 156 controls and 211 cases who were (+/-) or (+,+). They may not have had an adequate sample size to detect a significant association for *GSTT1*. For *GSTP1*, they had even fewer subjects which would not have allowed the ability to detect an association. In the Pabna population, we reported that individuals with the GG genotype (Val/Val) had a 1.86 increased odds of skin lesions compared to those individuals with the *GSTP1 AA *genotype (OR 1.86 (95%CI 1.15–3.00). Ghosh et al reported having 3 cases and 3 controls with the (Val/Val) or GG genotype so they lacked the power to detect the association[43]. Our results are at odds with this population, however as with all studies of single-nucleotide polymorphisms there may be other polymorphisms that are of importance that are in linkage disequlibrium and that may be responsible for some of the observed effects. It has been hypothesized that *GSTP1 *activity can compensate for the absence of *GSTM1 *activity[44]. Our study in Pabna, Bangladesh was an ethnically homogenous population of a much larger sample size than the previous study. Our results were also limited by sample size when analysis was stratified on exposure and genotype for case control status for *GSTP1 GG *due too few subjects in the high exposure category. However, *GSTP1 GG *was statistically significant overall for skin lesions, as well as in the low exposure drinking water strata. Despite these limitations, significant differences in risk of skin lesions associated with main effects of *GSTT1 *and *GSTP1 *genotypes.

## Conclusion

While we did not detect statistically significant interactions on the multiplicative or additive scale between arsenic level and *GSTT1, GSTM1 *and *GSTP1 *polymorphisms, we report that the main effects of *GSTT1 wildtype *and *GSTP1 GG *appear to be associated with increased risk of skin lesions. A larger sample size would allow a better investigation of the effect of the *GSTP1 *polymorphism at higher arsenic exposure. There was no evidence of effect modification of *GST *polymorphisms and arsenic concentration of drinking water on risk of skin lesions. Further work is required to characterize the potential mechanisms related to arsenic metabolism and *GSTT1 *and *GSTP1 *polymorphisms.

## Abbreviations

GST Glutathione S-transferase, GSH Glutathione, As Arsenic, Monomethylarsonic acid MMA (V), Monomethylarsonous Acid MMA (III), Dimethylarsinic acid DMA (V), multidrug resistant protein transporters (MRP), reactive oxygen species (ROS), LRT Likelihood Ratio Test, OR Odds Ratio, Interaction Contrast Ratios (ICR), Body Mass Index (BMI), Deoxyribonucleic acid (DNA), JNK, c-Jun NH_2_-terminal kinase.

## Competing interests

The author(s) declare that they have no competing interests.

## Authors' contributions

KMM was responsible for preparation of the manuscript, analysis of the data, and was involved in lab work and sample preparation.

LR was responsible for study design, statistical consultation and manuscript editing.

AH was involved in statistical consultation.

PW was involved in statistical consultation and manuscript editing.

DPM was involved in statistical consultation and manuscript editing.

QQ, MR, and GM were responsible for study coordination in Bangladesh, questionnaire data collection, and collection of samples and data entry.

TS was responsible for exposure methods and editing of manuscript.

EG was responsible for dermatologic consultation regarding skin lesions.

LS was responsible for overseeing and conducting genotyping and ensuring quality of data.

DCC was the study PI, responsible for study design and management, manuscript editing.

All authors approve of the manuscript.
